# Real-time grading method of tunnel surrounding rock based on image recognition

**DOI:** 10.3389/frai.2026.1766828

**Published:** 2026-02-05

**Authors:** Yihuan Xiao, Hao Yuan, Qingye Shi, Zemin Qiu, Liao Tang, Yihua Yu, Yabin Li, Yin Pan, Qinghua Xiao

**Affiliations:** 1 School of Civil Engineering, Southwest Jiaotong University, Chengdu, Sichuan, China; 2 Sichuan Electric Power Design and Consulting Co., Ltd., Chengdu, China

**Keywords:** image processing, machine learning, ShuffleNetV2, surrounding rock classification, tunnel engineering

## Abstract

To enable rapid, accurate grading of tunnel surrounding rock during construction, we propose a real-time grading method that integrates image processing with lightweight deep learning. We developed an automated pipeline that combines image-processing techniques and machine-learning algorithms to extract and classify characteristic parameters of tunnel surrounding rock, enabling real-time monitoring and classification at the tunnel palm surface. The study demonstrates that: (1) Following the proposed image-acquisition standards for rock and tunnel palm surfaces, images are converted to grayscale, denoised, enhanced, and normalized, which facilitates efficient and accurate extraction of structural features and improves the precision of classification parameters; (2) An optimized lithology identification and classification model was built, and a rock-hardness, strength, and integrity sensing approach based on the ShuffleNetV2 convolutional neural network was introduced to achieve real-time surrounding-rock grading. On an engineering site, the method attains 85% accuracy for lithology classification, 75% for rock-mass integrity, and 80% for overall surrounding-rock grade, confirming its feasibility and practical value. These results offer theoretical insight and engineering utility for the scientific evaluation of tunnel surrounding-rock grade.

## 1 Introduction

In recent years, the scale of underground engineering and tunnel construction has expanded rapidly, imposing greater challenges and higher technical standards on tunnel works. Consequently, traditional construction methods can no longer satisfy current demands for quality and schedule. As the foundation for tunnel design and construction, surrounding rock classification strongly influences both construction quality and progress. Consequently, achieving dynamic classification of tunnel surrounding rock has become a central research focus in geotechnical engineering.

Numerous scholars have investigated surrounding rock classification methods from both qualitative and quantitative perspectives. Zhuang et al. (2024) applied the state-of-the-art robust CNN model (EfficientNet) to tunnel wall image recognition and combined it with transfer learning to further improve the versatility, accuracy and efficiency of the deep learning model, ultimately achieving an accuracy of 89.96%. Vutukuri et al. (1974) employed parameters such as rock mass integrity and rock hardness as evaluation indicators for surrounding rock classification. Williamson (1984) introduced a rock classification system based on fundamental indicators, including the degree of rock weathering, rock strength, discontinuity degree, and density. Wang et al. (2020) employed the RMR, GSI, BQ, and HC methods to classify the quality of surrounding rock, utilizing on-site geological descriptions, drilling tests, and laboratory rock mechanics test results. Ma J. et al. (2023) and Ma X. et al. (2023) developed a refined prediction model for tunnel surrounding rock classification based on extension theory, integrating various geological methods, including geological mapping, ground-penetrating radar, tunnel seismic prediction, and advanced horizontal drilling. He et al. (2020) introduced a classification method for surrounding rock that accounts for the size effect of tunnel excavation spans and the presence of unfavorable geological formations. Wu et al. (2020) proposed a stability classification model for surrounding rock in underground engineering, utilizing conceptual lattice and TOPSIS, which is based on five indicators: rock quality grade, saturated uniaxial compressive strength, integrity coefficient, longitudinal wave velocity, and fractal dimension. Tan et al. (2022) employed the discrete element method to simulate the rock-breaking process of a pneumatic rock drill and, in conjunction with field data, established a standard database for the dynamic classification of surrounding rock. Nevertheless, the above conventional classification approaches are constrained by protracted parameter acquisition and the subjective nature of pivotal indicators.

Consequently, numerous scholars have adopted intelligent perimeter rock grading methods for their research, significantly reducing both time and economic costs while yielding substantial results. Li et al. (2018) and colleagues introduced a reliability analysis theory grounded in the national standard BQ method and employed the Monte Carlo method to classify surrounding rock based on evaluation indices such as rock toughness and integrity. Shi et al. (2014) proposed an over-optimized classification method utilizing fuzzy hierarchical analysis and tunnel seismic prediction to achieve precise predictions of surrounding rock classification. Additionally, Ma J. et al. (2023) and Ma X. et al. (2023) developed an intelligent surrounding rock classification method alongside a tunnel information management system, enabling real-time and accurate predictions of surrounding rock classification. Ma et al. (2022) proposed a probabilistic prediction method based on a Bayesian network for classifying tunnel surrounding rock quality using incomplete data. They validated their approach with data collected from 286 cases across 10 tunnels and found that the proposed method demonstrates high accuracy in predicting sample results despite data incompleteness. Shi et al. (2024) employed an integrated learning prediction model that combines XGBoost with Optuna for hyper-parameter optimization and real-time identification of perimeter rock classes. Xue et al. (2019) developed a perimeter rock classification model that incorporates five key factors: uniaxial compressive strength, rock integrity coefficient, softening coefficient, joint surface coefficient, and groundwater. This model was constructed using principal component analysis and the Ideal Point Method. Liu et al. (2020) proposed an integrated learning model that combines the classification and regression tree with the AdaBoost algorithm for predicting perimeter rock classification based on tunnel boring machine digging parameters. Zhao et al. (2022) employed ten supervised machine learning algorithms to develop an intelligent perimeter rock classification model and software system driven by drilling parameters. Song et al. (2023) utilized computerized perimeter rock data collected by a rock drilling cart, applying SMOTE, Random Forest, and XGBoost algorithms to achieve automatic classification and dynamic prediction of perimeter rock at the digging face.

Image recognition, a significant factor in the classification of enclosing rock, has been extensively examined by numerous researchers. Reid and Harrison, 2000 introduced a semi-automatic method for detecting discontinuous traces in grayscale digital images and conducted a preliminary analysis of palm surface information. Leu and Chang, 2005 utilized digitized image processing technology to extract rock cracks, subsequently establishing a three-dimensional model that illustrates the internal conditions of the tunnel based on the extracted crack information. Leng et al. (2021) employed traditional image processing techniques to perform image segmentation and edge fitting on geological information, including rock crevices and cracks identified through edge detection, thereby achieving more precise rock trace information. Liu and Wang (2023) employed transfer learning techniques to train extensive rock image datasets, facilitating the automatic identification and classification of the properties of tunnel surrounding rocks. Huang and Chen (2023) developed a fine grading model for surrounding rock by integrating heterogeneous data from multiple sources, utilizing a database of photographic images of excavation surfaces in rock tunnels, alongside on-site measurements, data statistics, intelligent algorithms, and numerical simulations. Chen et al. (2024) gathered over 7,000 images of tunnel palm surfaces from various tunnel project sites and constructed convolutional neural network classification models, including the VGG, ResNet, DenseNet, GoogleNet, and InceptionV3, to achieve intelligent identification of grading features in tunnel enclosing rocks and enable visualization analysis. Sun et al. (2023) developed a quality evaluation method and standard for broken rock bodies based on the degree of rock fragmentation and occlusion. This method employs digital imaging technology, image processing software, and multi-factor analysis to assess broken rock bodies in large underground caverns. Additionally, they proposed a damage mode and safety guidelines for the surrounding rock. However, these methods lack sufficient accuracy in extracting critical information regarding the grading of enclosing rock, such as joints and fissures in the palm surface at construction sites. The accuracy of image feature extraction diminishes, particularly under conditions of dust and low light, and the high complexity of the model poses challenges for deployment on mobile.

In summary, despite the extensive research on tunnel surrounding rock classification and image analysis, most existing studies concentrate on enhancing classification methods or individual image processing techniques. There is a notable absence of systematic investigations into the deep integration of machine learning with multi-scale image characterization, particularly regarding real-time applications in engineering. This gap is especially pronounced in complex construction environments, where developing a high-precision, deployable, and interpretable intelligent classification method for surrounding rocks based on image recognition remains a significant challenge.

In response to this challenge, this paper proposes a real-time classification method for surrounding rock that integrates a lightweight deep network with multi-scale feature analysis. This approach optimizes the network structure and parameter scale, achieving a balance between recognition accuracy, real-time performance, and engineering deployability. Additionally, by enhancing the image processing algorithm, the method accurately extracts geometric features of fissures in complex environmental conditions, addressing challenges in feature extraction, model deployment, and standardization in engineering applications. Furthermore, this study integrates automatically detected lithology and integrity parameters into the existing BQ grading system, enabling intelligent perimeter rock grading compliant with engineering standards. The proposed method is ultimately validated through real-world tunneling cases. The results indicate that the method demonstrates strong stability, accuracy, and engineering applicability, thereby offering reliable technical support for the safe, efficient, and economical construction of tunneling projects.

## 2 Tunnel feature parameter acquisition and processing

**Figure 1:**
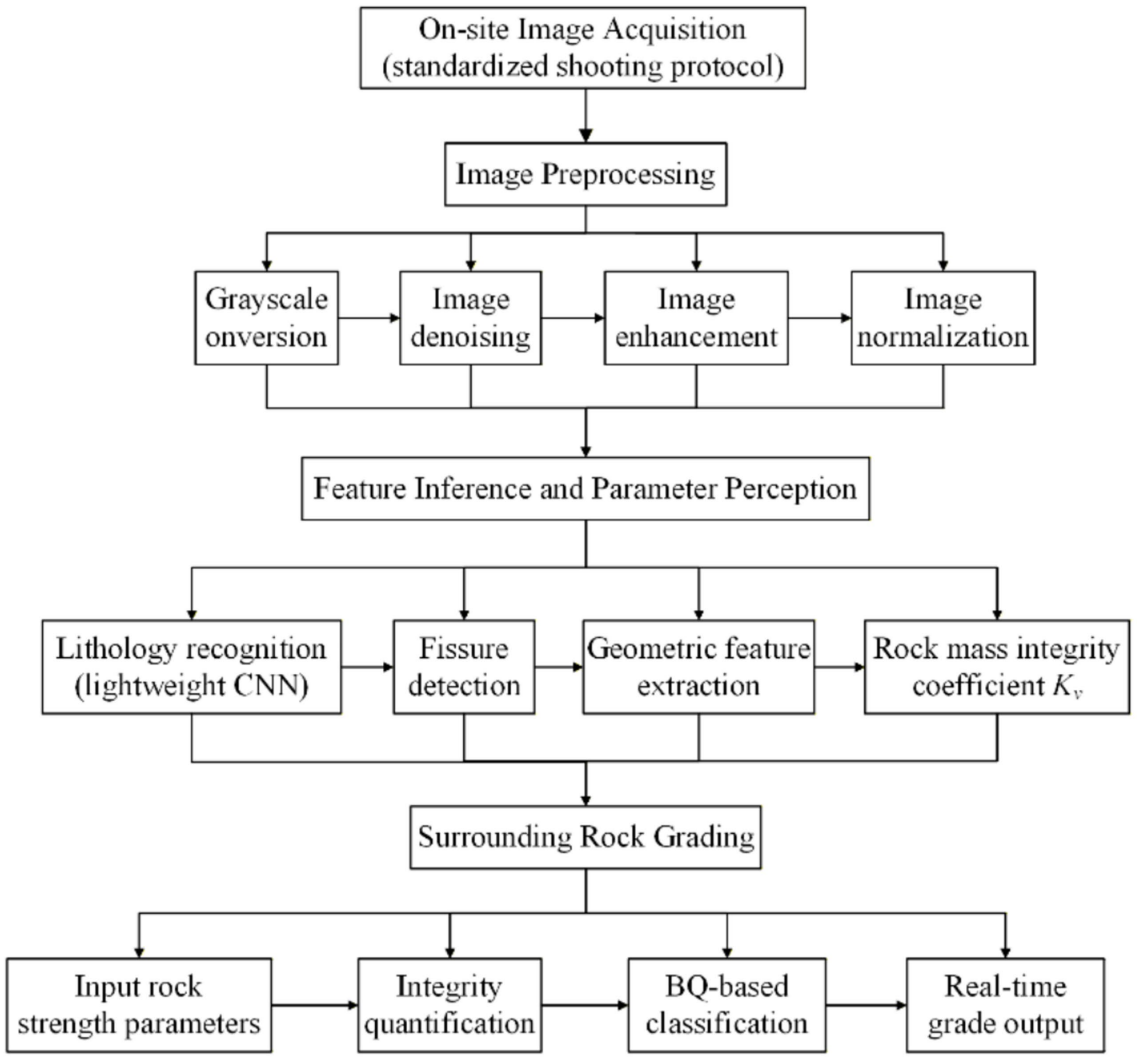
Flowchart of the real-time intelligent classification method for tunnel surrounding rock.

Tunnel palm surface image recognition plays a crucial role in contemporary tunneling projects. By acquiring and recognizing images of the palm surface, it is possible to obtain timely and accurate information regarding the state of the surrounding rock, thereby enabling real-time monitoring of tunnel construction and enhancing safety. This chapter examines and establishes the acquisition requirements and standards for various key feature parameters. Additionally, OpenCv, an open-source computer vision library widely employed for image processing, is utilized to preprocess the captured images. The entire processing flow is shown in Figure 1.

### 2.1 Rock image acquisition standards

Rock hardness is primarily influenced by lithology, which can be identified through rock imaging. Consequently, rock images serve as a crucial characteristic parameter of the surrounding rock, significantly contributing to lithological classification. The fundamental basis for determining rock lithology includes its structural characteristics, tectonic type, and color, among other factors. In the field of engineering geology, rocks are typically categorized into three groups based on their genesis, with their respective characteristics presented in Table 1.

**Table 1:** The geological characteristics of the three major types of rocks.

Geological features	Rock categories		
	Igneous	Sedimentary	Metamorphic
Genesis	Formed directly from high-temperature molten magma through magmatic processes	Formed from the weathering products of pre-formed rocks through diagenetic processes such as compaction and cementation.	Formed by metamorphism of pre-existing igneous, sedimentary, and metamorphic rocks
Texture	Crystalline granular and mottled structures	Characterized by a clastic, muddy, and bioclastic structure	Metamorphic structure
Tectonics	Massive, flow-like, pore-like, and amygdaloidal structures	Layered structure	Multiple foliated structures

Utilizing the structural and tectonic characteristics of rocks, along with additional factors such as rock color, computer image recognition technology can efficiently and accurately identify lithology. Consequently, it is essential to understand the requirements for rock image acquisition and to establish corresponding standards to ensure precise lithological determination. Based on this premise, the following acquisition standards are proposed: (1) Rock images must be captured using mobile smart devices or cameras with a minimum resolution of 20 megapixels. When photographing rocks within a tunnel, incandescent lamps should be employed for supplemental lighting. (2) On-site image data acquisition may occur during two specific time periods: 1. following the completion of tunnel blasting and after a ventilation period of 30 min; 2. after the discharge of slag has been finalized. (3) Select rocks exhibiting prominent structural features as subjects for imaging. Prior to capturing images, clean the surfaces of the chosen rocks to eliminate any stains or soil, thereby minimizing interference and noise in the resulting images. (4) Position the camera in alignment with the rock to ensure clarity in the images, and capture the same rock mass from multiple angles, obtaining 3 to 5 images that highlight its structural and tectonic characteristics. (5) Following image acquisition, document the time, tunnel, and mileage associated with the capture, and assess the quality of the rock photographs for classification and storage, facilitating subsequent research and analysis.

### 2.2 Tunnel palm surface image collection standard

The integrity of surrounding rock during tunnel construction is primarily assessed through the condition of the palm surface, which can be evaluated using palm surface imagery. This imagery contains critical information regarding structural surfaces, rock stratification, faults, joints, and cracks, all of which are essential for analyzing the integrity of the surrounding rock. Therefore, it is imperative to establish specific acquisition standards for palm surface image collection to enhance the quality of the images obtained. The acquisition requirements are as follows: (1) When capturing images of the palm surface, it is essential to ensure adequate brightness, with no obstruction from construction workers or equipment. This will facilitate high gray values and pronounced crack gradient variations during image processing. (2) Select a time when dust content is low to capture images of the palm surface, thereby ensuring minimal image noise. (3) Conduct forward photography on the images to prevent any visual distortion in the captured results. (4) In the presence of groundwater, choose an appropriate acquisition time, utilize waterproof equipment, and preprocess the images to mitigate the adverse effects associated with groundwater.

### 2.3 Image preprocessing

The quality of captured images at excavation sites is frequently compromised by light, dust, and construction equipment, necessitating preprocessing to aid in extracting structural information from rock and palm surfaces. In this study, we employ Python software to enhance image quality through grayscaling, noise reduction, enhancement, and normalization.

#### 2.3.1 Image grayscale

**Figure 2:**
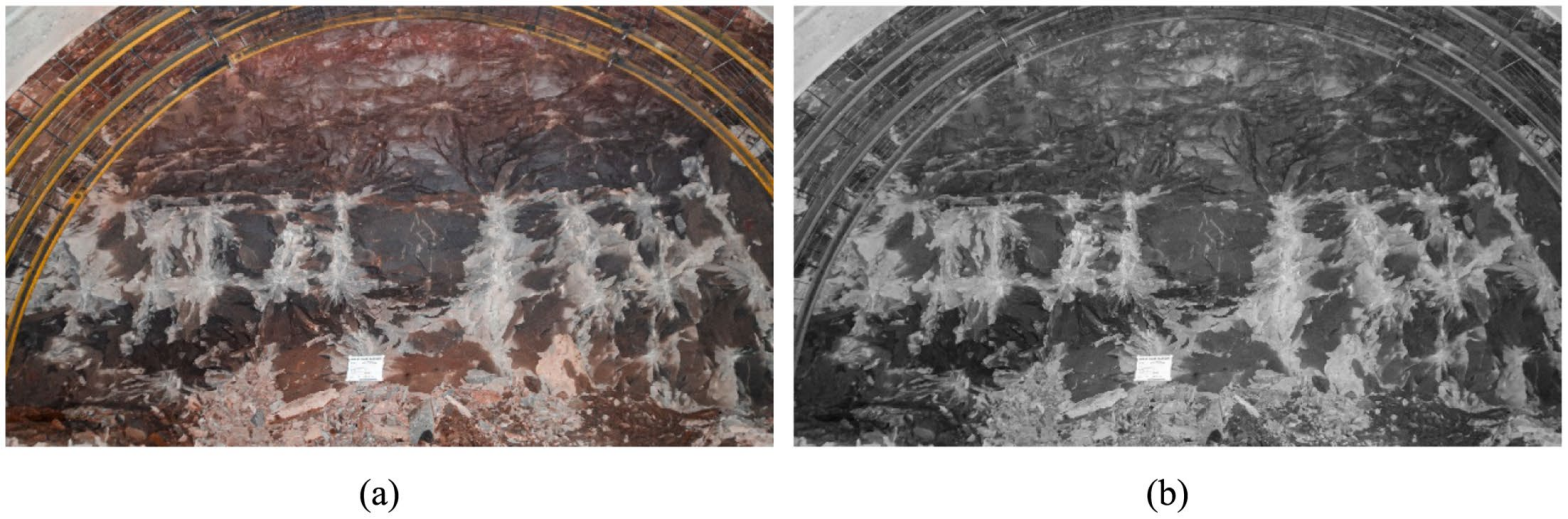
Image grayscale processing: **(a)** before graying; **(b)** after graying.

Image grayscaling involves converting a color graphic into grayscale through a series of algorithms. Images captured by cell phones or digital cameras are typically in color; however, the presence of color and lighting factors complicates the extraction of features such as joints and cracks on the palm surface. This complexity results in a significantly larger computational load compared to processing grayscale images. Therefore, prior to feature extraction in palm surface images, it is essential to convert the images to grayscale. Subsequent analysis focuses on the grayscale images, which enhances data processing efficiency and reduces algorithmic complexity. Additionally, grayscaling mitigates issues related to color reflections and shadow interference. The results of the image processing are illustrated in Figure 2.

#### 2.3.2 Image noise reduction

**Figure 3:**
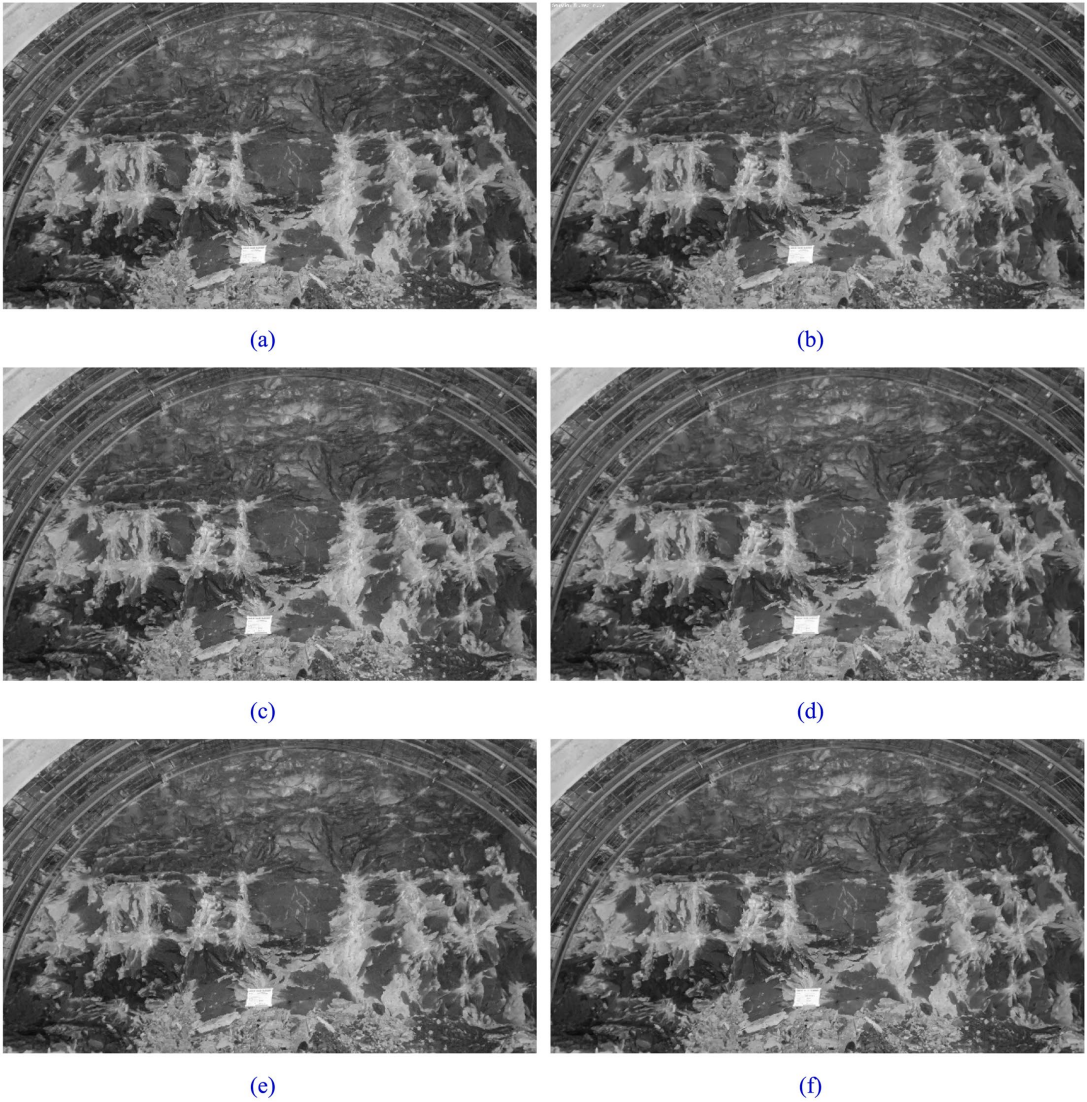
Gaussian filtering, median filtering, and non-local means noise reduction processing: **(a)** before Gaussian filtering denoising; **(b)** after Gaussian filtering denoising; **(c)** before median filtering denoising; **(d)** after median filtering denoising; **(e)** before NL-means denoising; **(f)** after NL-means denoising.

In this study, we contrast three frequently employed processing techniques: Gaussian Filtering, Median Filtering, and NL-Means Denoising, to ascertain the most suitable noise reduction approach. The outcomes of the image processing for these three methods are presented in Figure 3.

Assessing the quality of images produced by the three aforementioned noise reduction methods is challenging when relying solely on visual inspection. Therefore, the assessment of image quality post noise reduction relies on Mean Square Error (MSE), Peak Signal-to-Noise Ratio (PSNR), and Structural Similarity Index (SSIM), as demonstrated in Table 2.

**Table 2:** Image noise reduction evaluation metrics.

Processing method	MSE	PSNR	SSIM
Gaussian filtering	11.87	37.39	0.92
Median filtering	18.98	35.35	0.86
NL-Means denoising	6.07	40.29	0.95

Table 2 reveals that NL-Means Denoising achieves the lowest MSE of 6.07, suggesting the closest resemblance to the original image. Additionally, NL-Means Denoising demonstrates the highest PSNR and SSIM values, indicating superior quality and structural similarity to the original image. The comparison highlights NL-Means Denoising as the most effective method for noise reduction. Consequently, this study employs the non-local mean denoising approach for image noise reduction.

#### 2.3.3 Image enhancement

**Figure 4:**
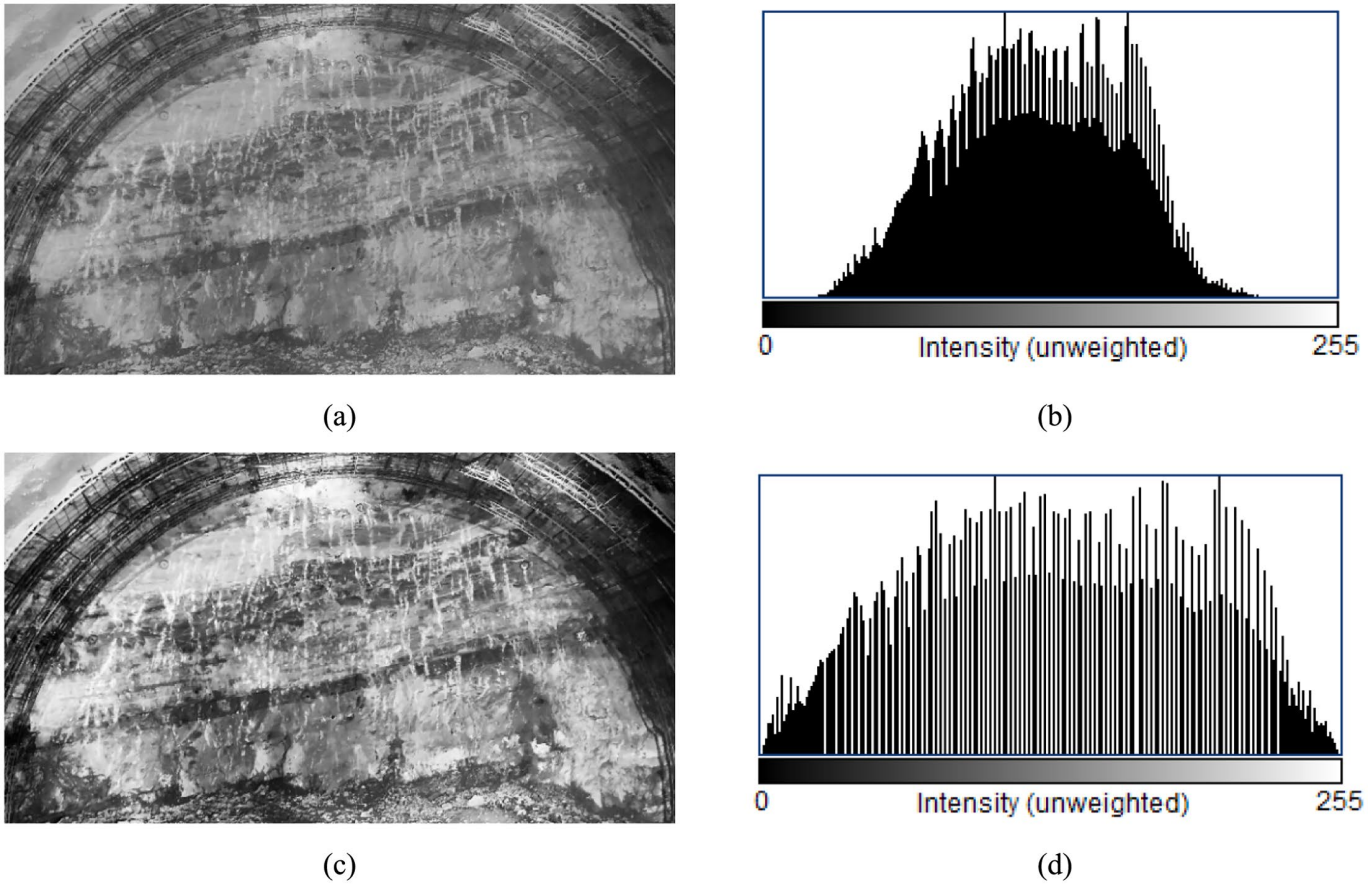
Tunnel palm surface histogram equalization: **(a)** tunnel palm surface before equalization; **(b)** the histogram before equalization; **(c)** tunnel palm surface after equalization; **(d)** the histogram after equalization.

The primary objective of image enhancement is to accentuate crucial information while diminishing irrelevant details to facilitate better comprehension, analysis, and visualization of the image. Enhancing rock and palm surface images involves improving the visibility of joints, fissures, as well as enhancing the texture and structural details within the image. In this study, histogram equalization technique is chosen for enhancing rock and palm surface images. The gray scale map of the palm surface undergoes histogram equalization using OpenCV, and the outcomes are illustrated in Figure 4.

**Figure 5:**
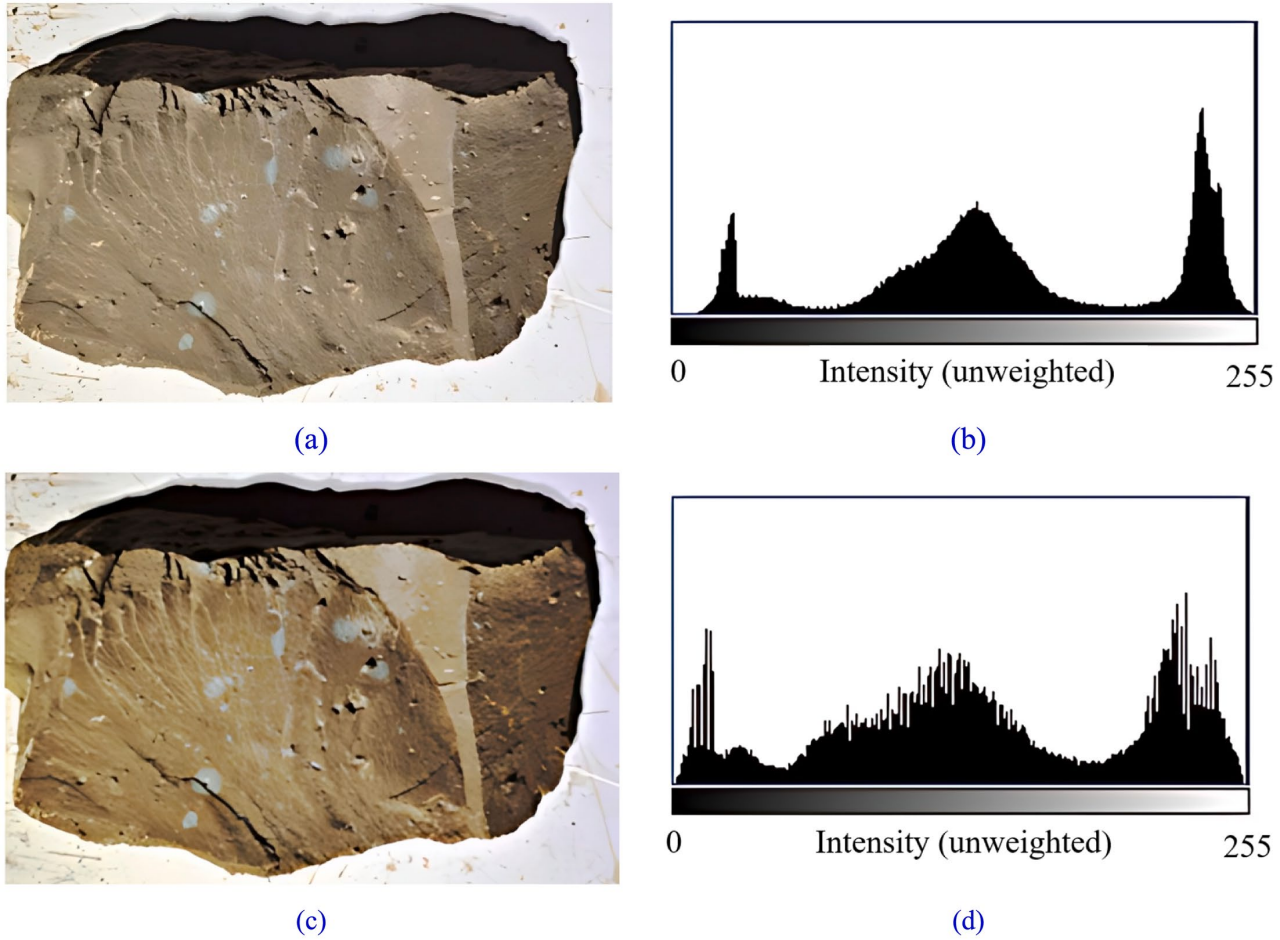
Histogram equalization of colored rock image: **(a)** tunnel palm surface before equalization; **(b)** the histogram before equalization; **(c)** tunnel palm surface after equalization; **(d)** the histogram after equalization.

The histogram equalization technique was originally applied to grayscale images; however, advancements in technology have enabled its extension to color image processing. The outcomes of processing rock color images are illustrated in Figure 5.

It can be seen from Figures 4, 5 that histogram equalization of the acquired rock and palm surface images enhances the overall contrast. This process clarifies details such as the joints and fissures of the palm surface and the texture of the rock. Additionally, the histogram indicates an expanded overall gray level range, with a more uniform distribution.

#### 2.3.4 Image normalization

The Min-Max Scaling method in deep learning facilitates image normalization through isometric scaling. This process involves linearly transforming the original image data to map it within the range of [0,1], thereby preserving the relative scales among the features, as computed in Equation 1.


$$X_{\mathrm{norm}}=\frac{X-X_{\min}}{X_{\max}-X_{\min}}$$ (1)

**Figure 6:**
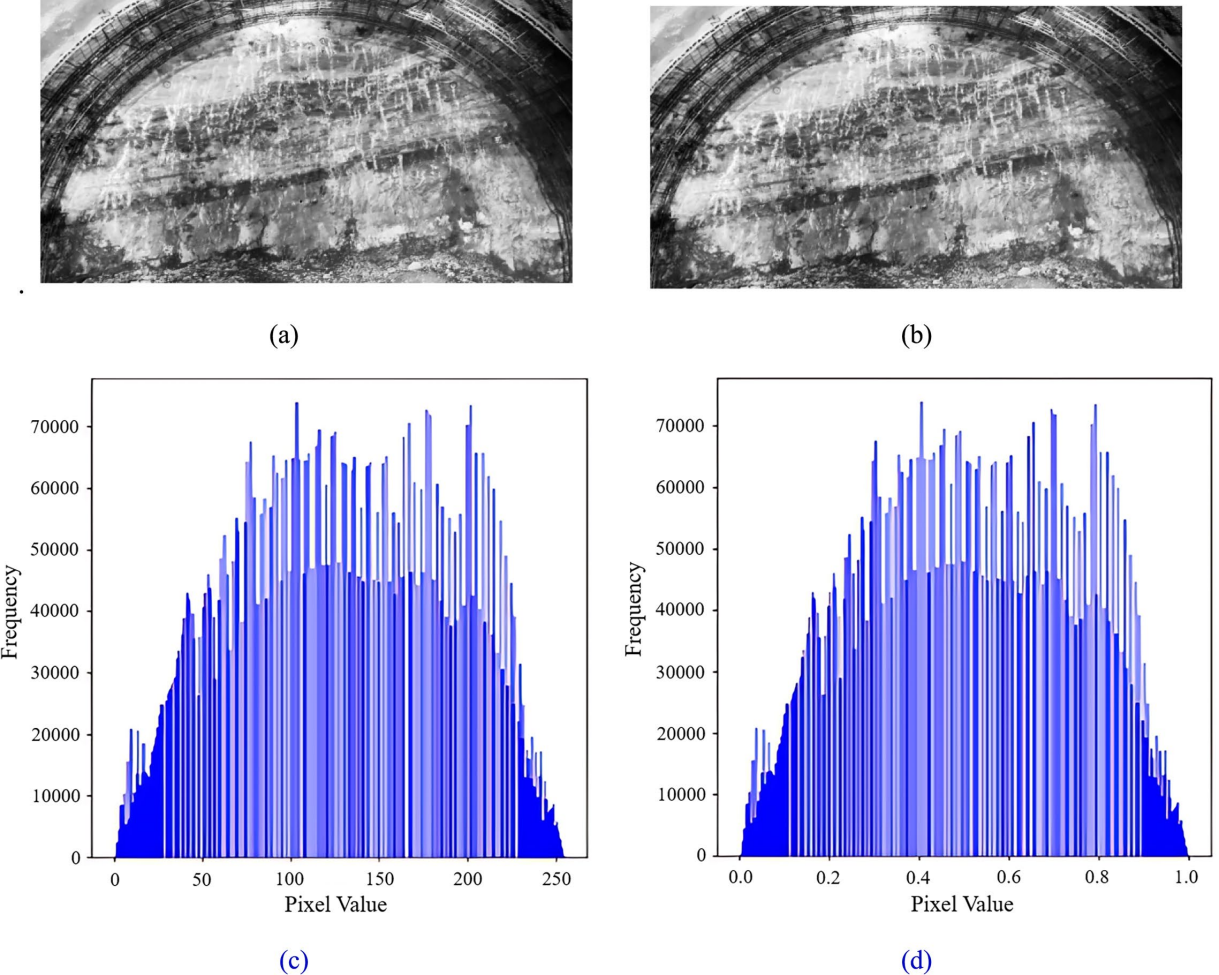
Tunnel palm surface image normalization: **(a)** tunnel palm surface image before normalization; **(b)** tunnel palm surface image after normalization; **(c)** histogram of the tunnel palm surface before normalization; **(d)** histogram of the tunnel palm surface after normalization.

**Figure 7:**
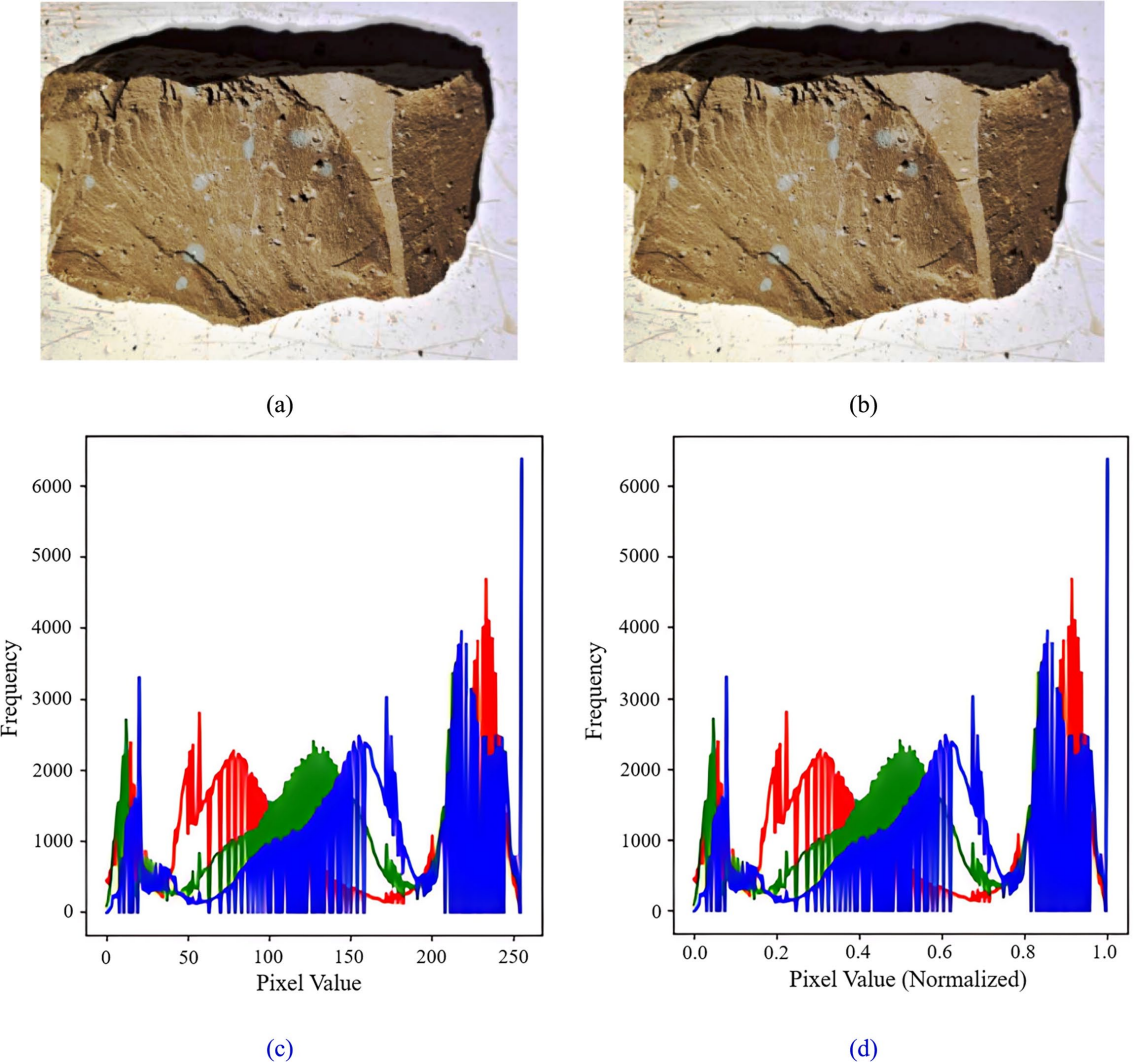
Rock image normalization **(a)** rock image before normalization; **(b)** rock image after normalization; **(c)** histogram of rock before normalization; **(d)** histogram of rock after normalization.

Where *X_norm_* is the normalized data, *X* is the original data, *X*_min_ and *X*_max_ are the minimum and maximum values of the data, respectively. The rock and tunnel palm surface images are normalized respectively, and their original images and corresponding histograms are shown in Figures 6, 7.

Figures 6, 7 illustrate that the distribution characteristics of the pixels in the rock and palm surface images remain unchanged following normalization. However, the pixel value range is reduced from [0, 255] to [0, 1]. This indicates that the normalization process preserves the information and features of the images while merely adjusting the scale of the pixel values. Furthermore, normalization facilitates faster convergence of the training model and enhances the model’s generalization capability.

## 3 Automatic perception and classification method of tunnel surrounding rock

The primary characteristic parameters of perimeter rock grading include the degree of rock hardness and the integrity of the rock body, both of which are critical for assessing the perimeter rock grade. Consequently, the rapid and precise acquisition of these key feature parameters is essential for real-time grading of surrounding rock during the tunnel construction phase. This chapter employs deep learning and image processing techniques to extract feature parameters. A ShuffleNet convolutional neural network model is developed to classify and recognize the properties of rock images. Additionally, image processing technology is utilized to analyze the palm surface images, enabling the extraction of structural characteristics and the identification of key feature parameters for enclosing rock grading.

### 3.1 Automatic perception of rock hardness

In the BQ method for perimeter rock classification, rock hardness serves as a critical index. When classifying surrounding rock, it is essential to not only qualitatively assess the rock’s hardness but also to determine its specific uniaxial saturated compressive strength. This section employs the ShuffleNet V2 convolutional neural network to classify rock images and identify lithology. The rock hardness is subsequently derived from the established mapping relationship between lithology and rock hardness for specific tunnels.

#### 3.1.1 Rock image dataset

The rock image samples in this study were acquired through three methods: field collection, laboratory acquisition, and web collection. The samples collected from the field primarily originated from specific railroad tunnel projects in Southwest China, specifically in Sichuan and Yunnan. Initially, rocks were gathered at the construction site following the image acquisition criteria outlined in a previous publication. A total of 1,010 rock sample photos were amassed, comprising 606 field-collected, 185 laboratory-acquired, and 219 web-collected images. Subsequently, the gathered rock images were classified and refined, resulting in 163 Class A photos (magmatic rocks), 681 Class B photos (sedimentary rocks), and 166 Class C photos (metamorphic rocks). Images with lens imperfections, blurriness, and intricate backgrounds were excluded, yielding 127 Class A images, 474 Class B images, and 129 Class C images, totaling 730 images. We employed a stratified random sampling approach to allocate the screened images, with 70% (510) assigned to the training set, 15% (110) to the validation set, and 15% (110) to the test set. This allocation aimed to maintain equal proportions of magmatic, sedimentary, and metamorphic rocks across the training, validation, and test sets, ensuring experimental reproducibility. Table 3 presents the distribution of rock photos across various lithologies.

**Table 3:** Classification and quantity of rock sample set.

Rock classification	Rock type number	Lithology classification	Number of pictures collected	Number after screening
Igneous	A1	Granite	67	52
	A2	Basalt	44	35
	A3	Andesite	52	40
Sedimentary	B1	Mudstone	210	135
	B2	Shale	134	95
	B3	Sandstone	157	112
	B4	Conglomerate	65	48
	B5	Limestone	115	84
Metamorphic	C1	Quartzite	55	44
	C2	Marble	57	42
	C3	Phyllite	54	43
Total			1,010	730

#### 3.1.2 Establishment of lithology identification model

**Figure 8:**
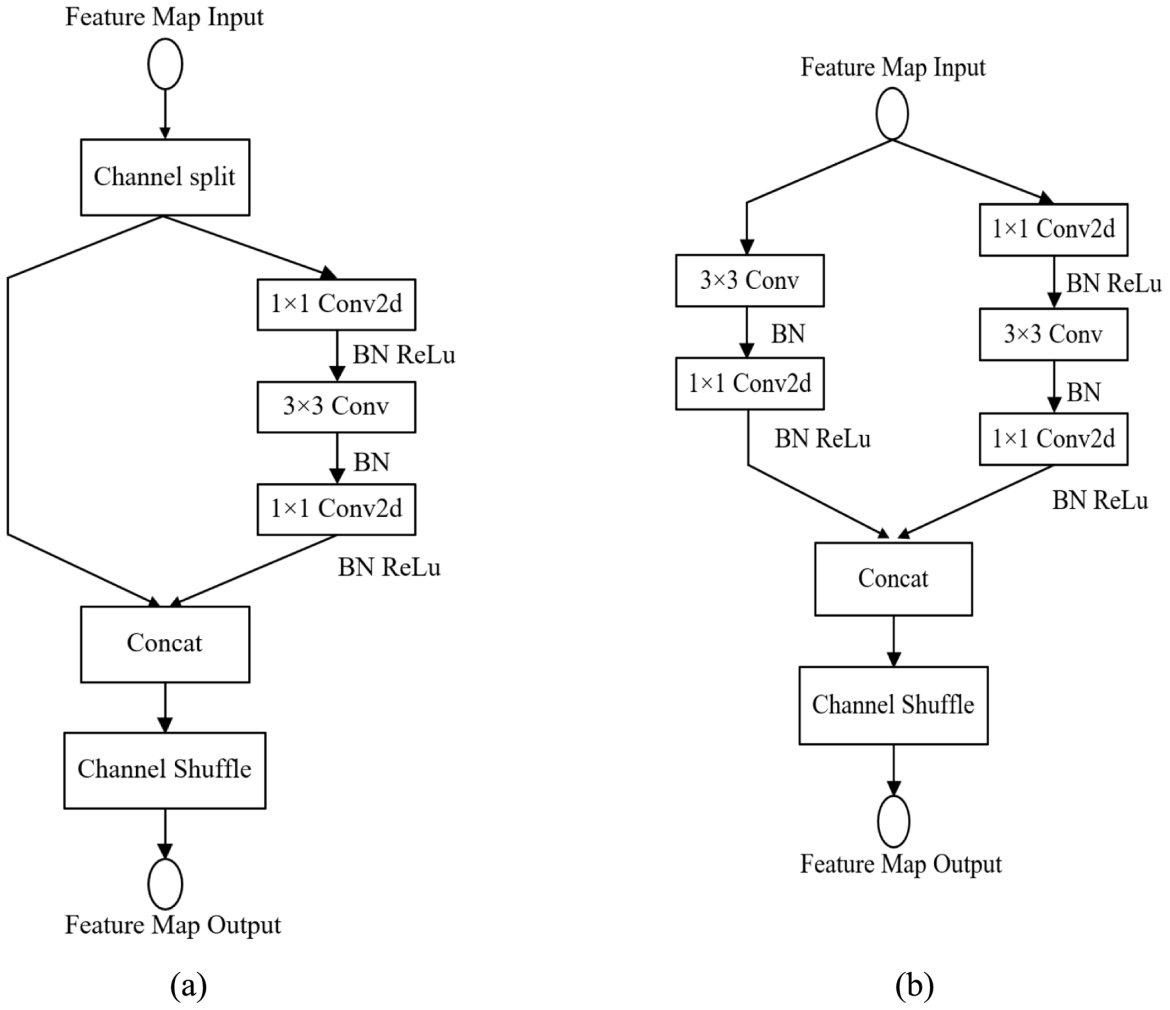
ShuffleNet V2 unit structure: **(a)** ShuffleNet V2 Unit1; **(b)** ShuffleNet V2 Unit2.

To develop the lithology recognition model, we conducted image lithology classification experiments utilizing 0.5×, 1.0×, and 1.5× grids within the ShuffleNet V2 framework, as detailed in Table 4. The rockiness classification model comprises a convolutional layer that incorporates (1) 3 × 3 convolutional kernels with Batch Normalization (BN) and Rectified Linear Unit (ReLU) activation functions, (2) a MaxPool layer with a stride of 2, and (3) three modular layers, each consisting of ShuffleNet V2 Unit1 and Unit2. The configuration of the ShuffleNet V2 Units is illustrated in Figure 8, with the ratio of Unit2 to Unit1 set at 1:3, 1:7, and 1:3, respectively. Additionally, the model includes (4) a 1 × 1 convolutional layer, followed by a GlobalPool layer, and concludes with a Fully Connected Layer (FC) that transforms the output from the GlobalPool layer into the final category prediction, employing a softmax activation function to yield the results.

**Table 4:** ShuffleNet V2 overall architecture.

Layer	Output size	Kernel size	Stride	Repeat	Output channels
Image	224 × 224				3
Conv1	112 × 112	3 × 3	2	1	24
MaxPool	56 × 56	3 × 3	2	1	
Stage2	28 × 28		2	1	48
	28 × 28		1	3	
Stage3	14 × 14		2	1	96
	14 × 14		1	7	
Stage4	7 × 7		2	1	192
	7 × 7		1	3	
Conv5	7 × 7	1 × 1		1	1,024
GlobalPool	1 × 1	7 × 7			
FC					11

Prior to model training, it is essential to establish the fundamental parameters and hyperparameters. This paper relies on relevant parameters derived from existing research (Gui, 2024; Yuan et al., 2017), incorporates necessary adjustments, and ultimately determines the model parameters, as presented in Table 5.

**Table 5:** ShuffleNet V2 model parameter settings.

Serial number	Symbol	Explanation	Value
1	Resize	Input image size	224 × 224
2	Epoch	Number of iterations	100
3	Learning-rate	Learning rate	0.001
4	Decay	Weight decay factor	0.0005
5	Batch-size	Number of training samples per batch	32

#### 3.1.3 Comparative analysis of test results of lithology identification model

The ShuffleNet V2 neural network model described in the previous section was employed to classify the lithology of 11 collected images representing magmatic, sedimentary, and metamorphic rocks. The training outcomes were subsequently analyzed and compared using various performance metrics.

The model was trained on the rock image dataset utilizing three distinct grid sizes: 0.5× grid, 1.0× grid, and 1.5× grid. The number of iterations was set to 100, and the hyperparameters were maintained at their default values. The results from the prediction set were analyzed comparatively using six performance metrics: Accuracy, Precision, Recall, F1 score, Total Parameters, and Model Size, as presented in Table 6.

**Table 6:** Comparison of model test results.

Grid-size	Accuracy	Precision	Recall	*F*_1_	Total-parameters	Model size
0.5×	87.58%	87.16%	85.08%	85.64%	353,067	1.35 MB
1.0×	86.97%	85.08%	84.69%	84.17%	1,264,879	4.82 MB
1.5×	86.57%	86.72%	84.21%	83.95%	2,489,899	9.5 MB

As shown in Table 6, the test accuracies for the 0.5× grid, 1.0× grid, and 1.5× grid models applied to the 1,460 test set of lithological images, yielding accuracies of 87.58, 86.97, and 86.57%, respectively. As the grid width increases, both the model’s classification accuracy and the number of computational parameters double, leading to an increase in model size. Consequently, the 0.5× grid of the ShuffleNet V2 neural network model demonstrates a significant advantage over the other models by maintaining a high accuracy rate of 87.58%. This outcome underscores the effectiveness of its lightweight design.

**Figure 9:**
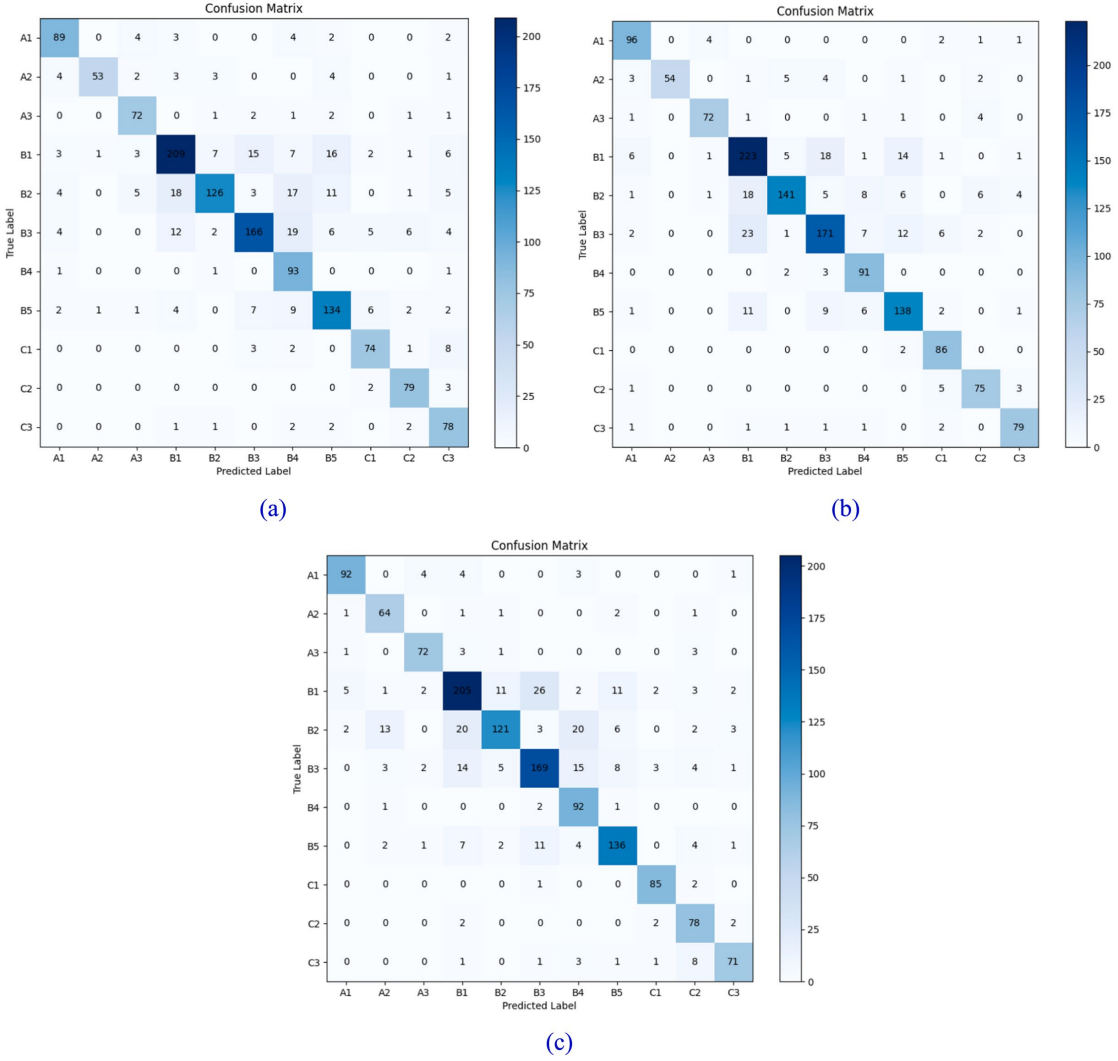
Confusion matrix of test sets with different grid widths: **(a)** 0.5× grid; **(b)** 1.0× grid; **(c)** 1.5× grid.

**Figure 10:**
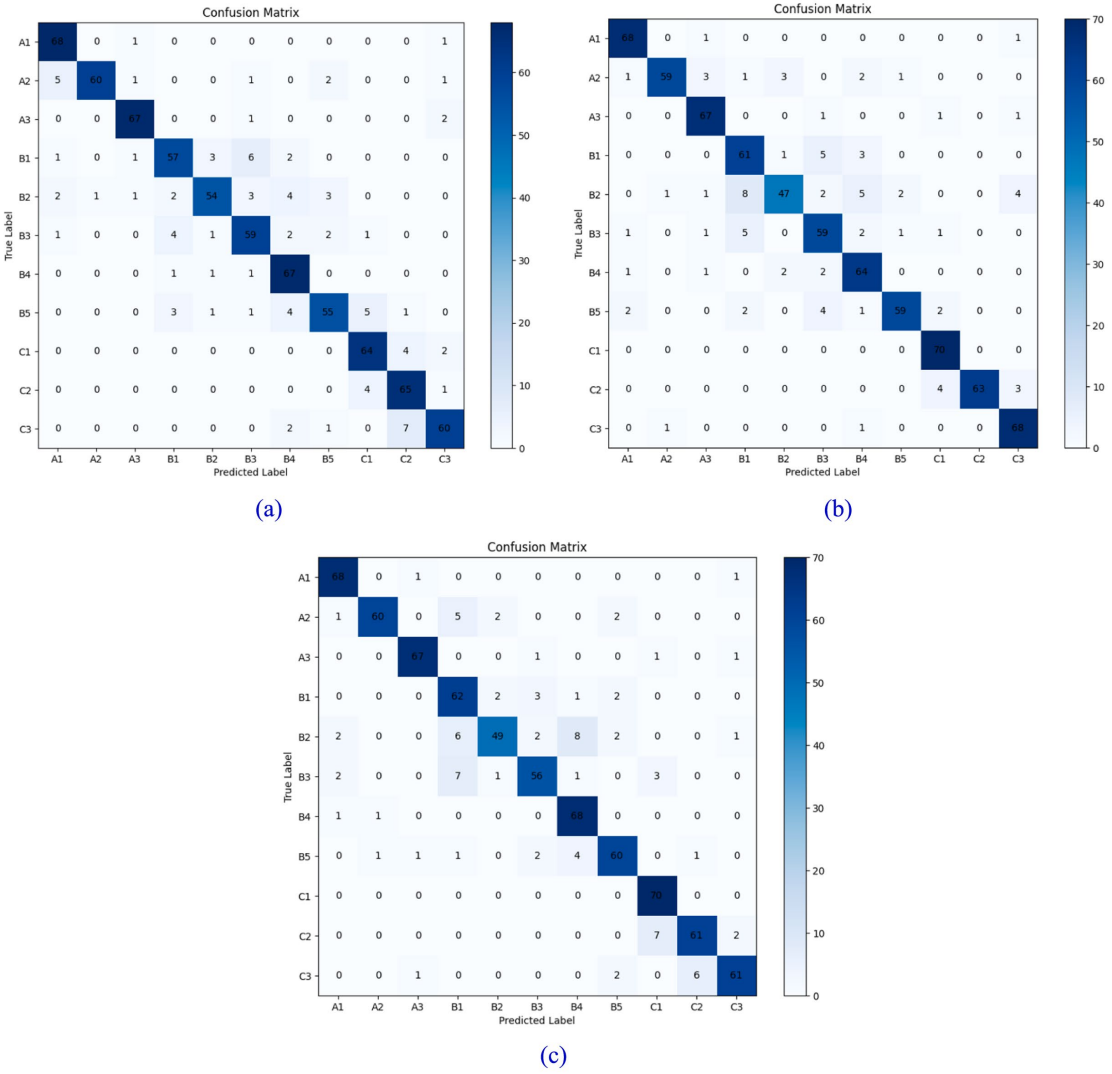
Confusion matrix of validation sets with different grid widths: **(a)** 0.5× grid; **(b)** 1.0× grid; **(c)** 1.5× grid.

To further validate the model’s accuracy, we analyze the classification results of the ShuffleNet V2 neural network for rockiness identification across three different grid sizes, utilizing the confusion matrix, as illustrated in Figures 9, 10. Each row corresponds to the model’s predicted categories, while each column represents the actual categories. The diagonal line indicates the count of instances where the predicted values match the actual values; thus, a higher value along the diagonal line signifies improved model performance.

A comprehensive comparison of the results from the three models reveals that the accuracy of the 0.5× grid model is slightly higher than that of the latter two grid models. Additionally, the number of computational parameters and the overall model size of the 0.5× grid model are significantly smaller than those of the other two models. The accuracy of the 1.0× grid model exceeds that of the 1.5× grid model by only 0.4%, yet the number of computational parameters in the 1.0× grid model is less than half that of the 1.5× grid model. Therefore, considering both computational accuracy and the number of parameters, this paper selects the 0.5× grid model for lithology classification and rock image recognition.

#### 3.1.4 Rock hardness

Rock hardness is ascertained by integrating rock lithology with the extent of weathering. The ShuffleNet V2 convolutional neural network, as previously discussed, can effectively classify the lithology of rock images. Subsequently, the geological investigation report of the particular tunnel allows for the determination of the weathering degree of the strata and the uniaxial saturated compressive strength of the corresponding rocks. Ultimately, based on Table 7, a comprehensive classification of rock hardness is conducted.

**Table 7:** Classification of rock hardness levels.

Category		*R*_c_ (Mpa)	Weathering degree	Lithology
Hard rock	Extremely hard rock	>60	Unweathered ~ slightly weathered	Granite, basalt, andesite, diorite, syenite, gneiss, quartzite, siliceous limestone, siliceous cemented sandstone or conglomerate, etc.
	Hard rock	60 ~ 30	Unweathered ~ slightly weathered	Marble, limestone, slate, calcareous cemented sandstone, etc.
			weak weathered	Granite, basalt, andesite, diorite, syenite, gneiss, quartzite, siliceous limestone, siliceous cemented sandstone or conglomerate, etc.
Soft rock	Slightly soft rock	30 ~ 15	Unweathered ~ slightly weathered	Phyllite, sandy mudstone, conglomerate, marl, shale, etc.
			weak weathered	Marble, limestone, slate, calcareous cemented sandstone, etc.
			strong weathering	Granite, basalt, andesite, diorite, syenite, gneiss, quartzite, siliceous limestone, siliceous cemented sandstone or conglomerate, etc.
	Soft rock	15 ~ 5	Unweathered ~ slightly weathered	Mudstones: mudstone, argillaceous cemented sandstone and conglomerate, etc.
			weak weathered	Phyllite, sandy mudstone, conglomerate, marl, shale, etc.
			Weak weathering ~ strong weathering	Marble, slate, limestone, calcareous cemented sandstone, etc.
	Extremely Soft rock	≤5	strong weathering	Mudstones: mudstone, argillaceous cemented sandstone and conglomerate, etc.
			fully weathered	All kinds of rocks

### 3.2 Automatic perception of surrounding rock integrity

The integrity of enclosing rock is one of the two fundamental indicators used for grading in the BQ method. This integrity is typically assessed based on parameters such as the number of nodal cracks, the area they occupy, and their size. In this study, the Canny algorithm is employed to extract image edges, followed by the application of the OTSU algorithm to determine the geometric dimensions of the nodal cracks. This process facilitates the measurement of crack size, the area occupied by the cracks, and other relevant characteristics, ultimately allowing for the evaluation of the surrounding rock’s integrity.

#### 3.2.1 Joint fissure edge detection

**Figure 11:**
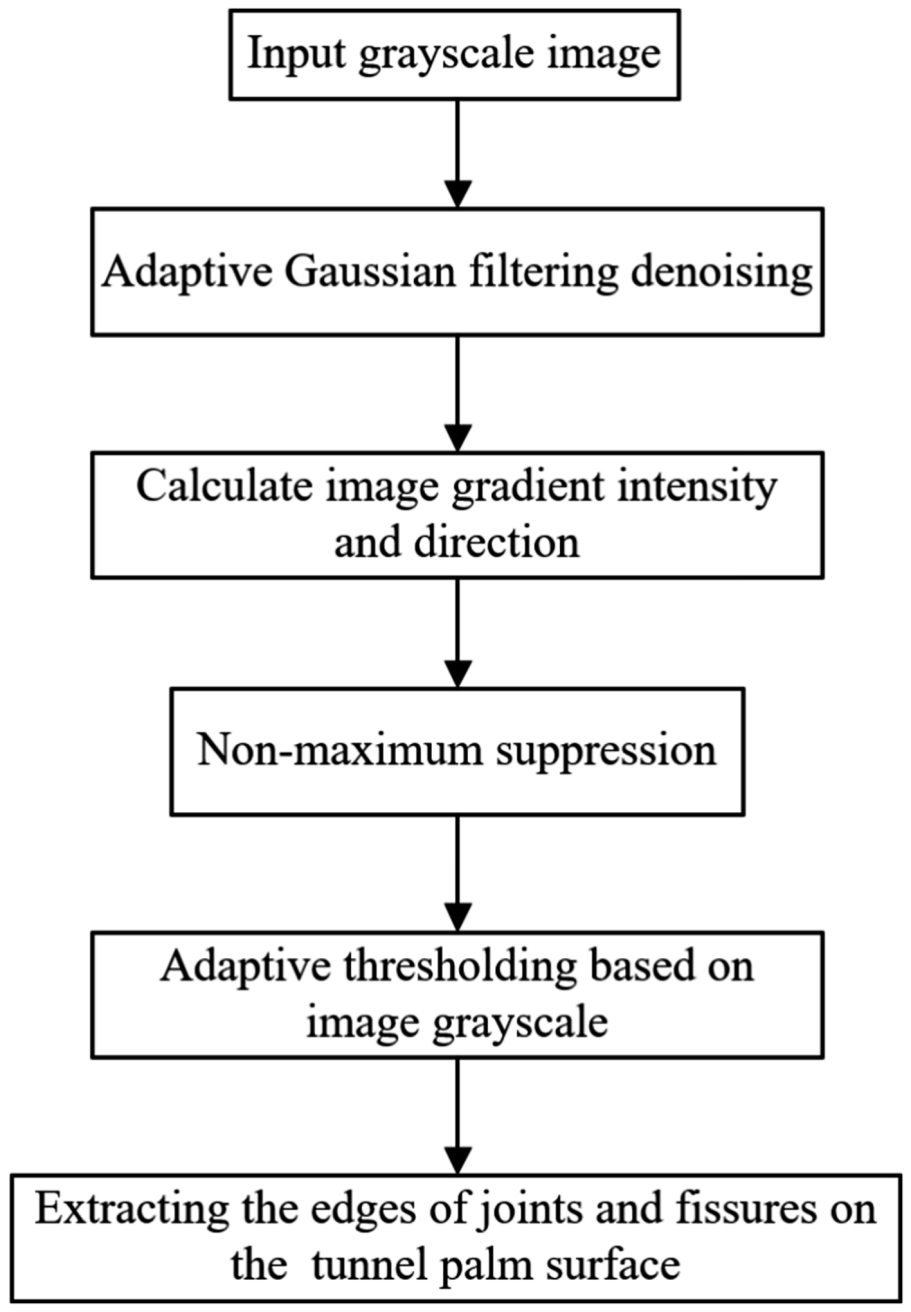
Flowchart of the improved Canny operator.

The Canny operator is employed for extracting nodal fissure features from tunnel palm surface images captured in the field. To enhance the nodal fissure edge extraction process, adaptive Gaussian filtering and adaptive thresholding techniques are integrated into the Canny operator. This integration allows for automatic adjustment of parameters based on the unique characteristics of each image, facilitating image feature recognition. The operational procedures are illustrated in Figure 11.

**Figure 12:**
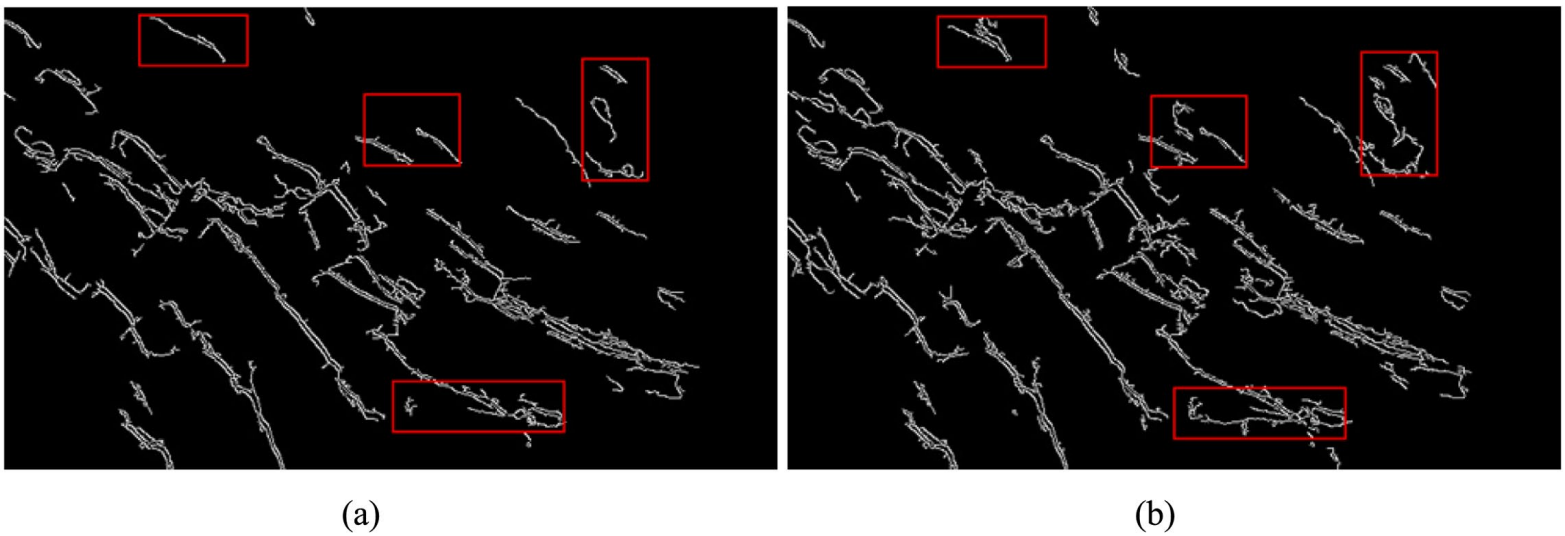
Comparison of adaptive Gaussian filtering and Canny detection: **(a)** basic Canny detection; **(b)** adaptive Gaussian noise reduction Canny detection.

To achieve automatic perception for image refinement, the Canny operator, along with adaptive Gaussian filtering, was employed under a consistent threshold for detecting nodal fissures on palm surface images. As illustrated in Figure 12, the adaptive Gaussian filtering Canny operator significantly diminishes noise interference, preserves a greater amount of image information, and enhances the identification of image features. This method demonstrates superior accuracy compared to traditional Canny operator detection.

**Figure 13:**
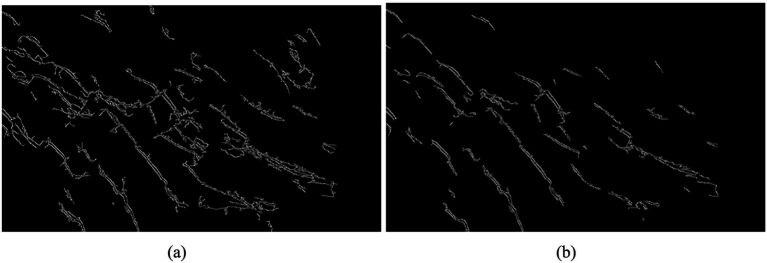
Comparison of adaptive threshold and Canny detection: **(a)** before using adaptive threshold; **(b)** after using adaptive threshold.

The threshold of the Canny operator significantly influences feature detection outcomes. A threshold that is too high may result in the loss of critical feature information, while a threshold that is too low can introduce pseudo-features. To enhance the adaptability of the Canny operator for identifying nodal cracks in palm surface images, the median gray value of the image is employed as the basis for threshold calculation. Subsequently, an adaptive thresholding statistical method is utilized for image segmentation. The results of this process are illustrated in Figure 13.

It can be seen from Figure 13 that the application of adaptive threshold detection results in the removal of certain fine edge features. However, this process significantly enhances the extraction of important features within the image, thereby improving the accuracy of nodal crack detection on the palm surface.

#### 3.2.2 Fissure body segmentation

The initial state of joint fissure serves as a crucial indicator for assessing the stability of adjacent rock formations. In this study, the OTSU method is employed to differentiate between the joint fissure and the rock matrix, thereby extracting the primary structure of the joint fissure. The procedural details are outlined below:

1. Calculate histogram.

Calculate the number of pixels at each gray level in the grayscale image after processing with the improved Canny operator.

1. Normalize the histogram.

Obtain the probability density function by the ratio of the number of pixels at each gray level to the total number of pixels.

1. Calculate the inter-class variance.

Let T represent the single-channel grayscale maximum. For any threshold *k*, the grayscale values of the image (denoted as t) are divided into two intervals, *C*_0_ and *C*_1_, where *C*_0_ is defined as $\left\{C_{0}|t<k\right\}$ and C1 as $\left\{C_{1}|k<t<T\right\}$ . Subsequently, the probability distributions of the two-pixel categories, *P*_0_(*k*) and *P*_1_(*k*), along with their respective mean values, *u*_0_(*k*) and *u*_1_(*k*), are computed. Finally, the interclass variance, *σ*^2^*_ω_*(*k*), is determined using the Equation 2:


$${\sigma^{2}}_{\omega }(k)=P_{0}(k)\times P_{1}(k)\times {\left[u_{0}(k)-u_{1}(k)\right]}^{2}$$ (2)

Where $P_{0}(k)=\sum_{i=0}^{k}p_{i},P_{1}(k)=\sum_{i=k+1}^{L-1}p_{i}$ , *L* is the number of gray levels; $u_{0}(k)=\frac{1}{P_{0}(k)}\sum_{i=0}^{k}p_{i}\times i$ , $u_{1}(k)=\frac{1}{P_{1}(k)}\sum_{k+1}^{L-1}p_{i}\times i$ .

1. Determination and application of optimal threshold.

**Figure 14:**
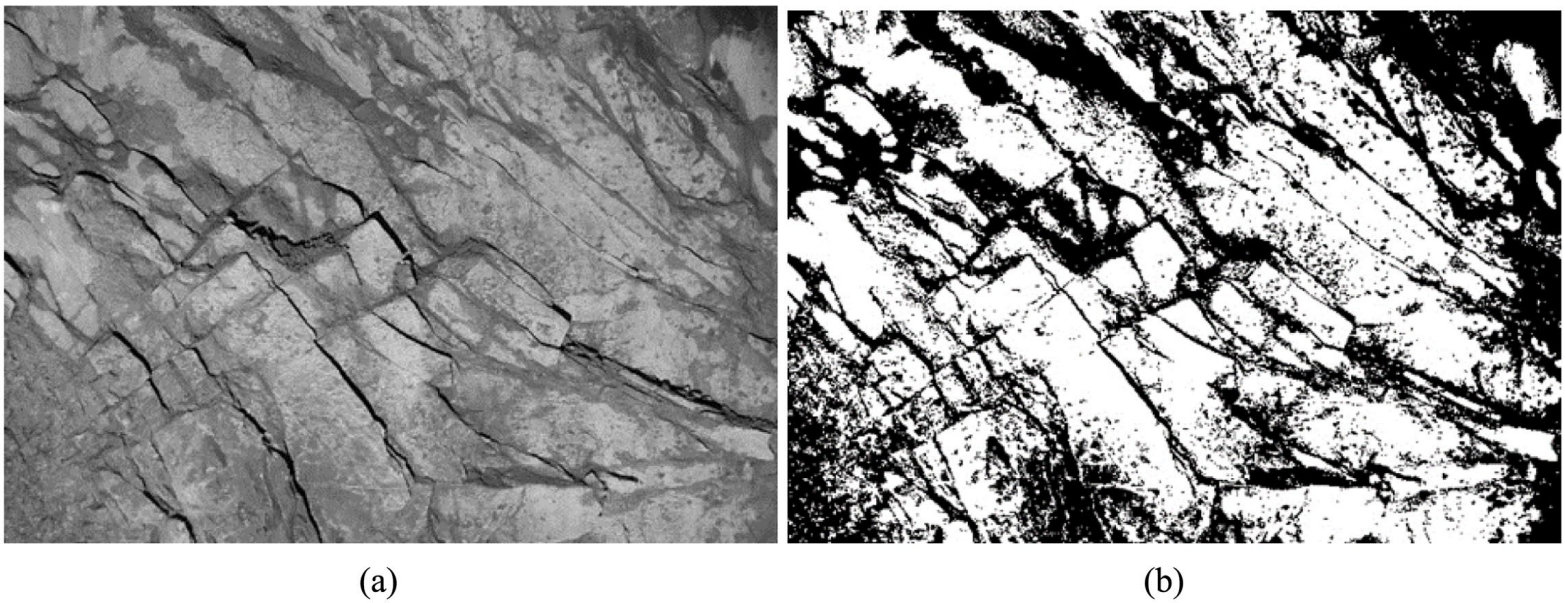
Traditional OTSU method for image segmentation: **(a)** grayscale image of joints on the tunnel palm surface; **(b)** image segmentation using the OTSU method.

The variances between different classes of threshold *k* are calculated for each value in the range [0, T]. When ${\sigma^{2}}_{\omega }(k)$ is maximized, this threshold is used for image segmentation. The image segmentation of the face of the tunnel using the OTSU method is shown in Figure 14.

**Figure 15:**
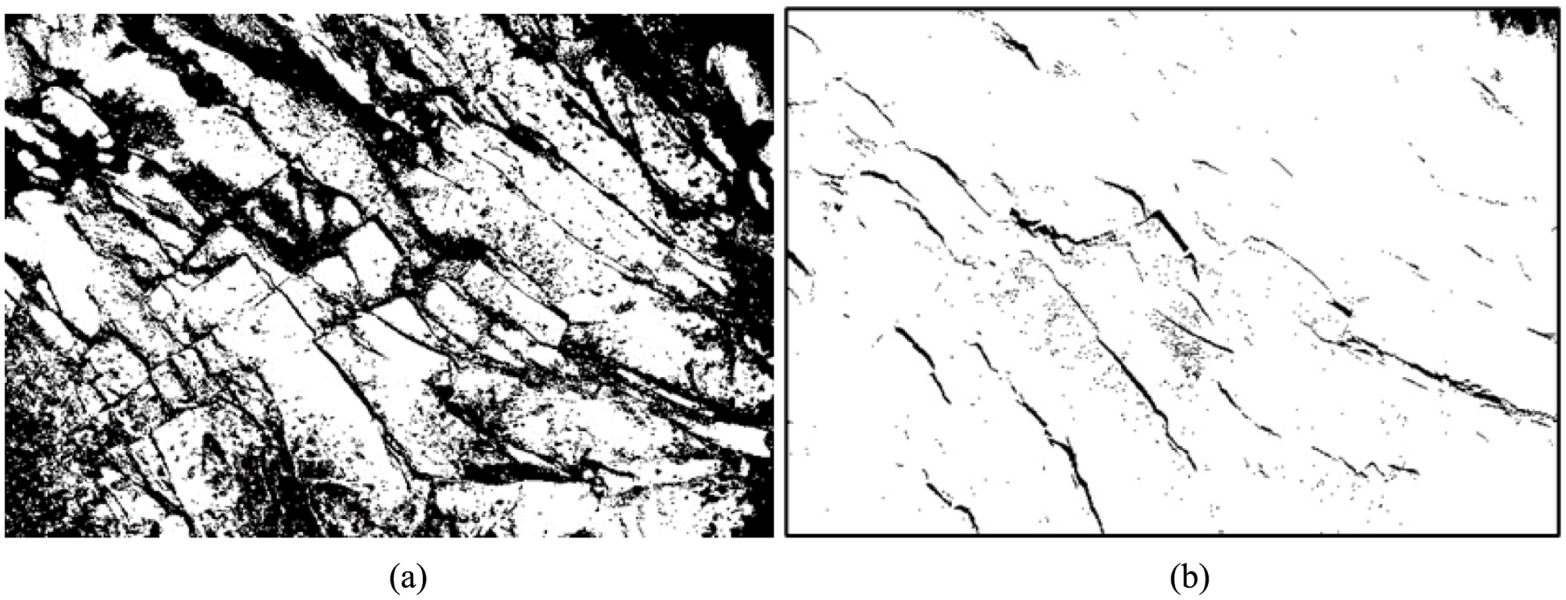
Improved OTSU method for image segmentation: **(a)** before improvement and **(b)** after improvement.

The presence of rock cover, combined with variations in light tilt and shooting angle, typically results in a low gray value for nodal fissures. As illustrated in Figure 14, the traditional OTSU method reveals significant overlaps between the segmented joint fissures and the surrounding rocks on the palm surface. This overlap contributes to a low accuracy in fissure recognition, resulting in substantial white and black blocks, as well as discontinuities in the segmentation diagrams. Consequently, improvements to this method are necessary. The traditional OTSU method categorizes the higher gray value part as the foreground and the lower part as the background when segmenting an image into front and back views. However, this approach is overly rigid. To address this limitation, our study introduces a dual-threshold segmentation method building upon the OTSU method. This method utilizes the optimal threshold value *k* from OTSU as a benchmark and defines a dual-threshold value range within the interval [*k* − 50, *k* + 50]: *T*_max_ = *k* + 50 and *T*_min_ = *k* − 50. Pixels falling above the high threshold or below the low threshold are designated as the background (white), while pixels within this range are identified as the foreground (black). This technique facilitates the extraction of nodal fissures, as illustrated in Figure 15.

#### 3.2.3 Integrity of surrounding rock

The assessment of rock integrity can be determined by the measured rock volume nodal number, *J_v_*. However, the *J_v_* value cannot be directly obtained through computer image processing. Consequently, the fissure ratio, *K*_s_, is introduced as an alternative metric for evaluating rock integrity, with the fissure ratio formula presented in Equation 3.


$$K_{s}=\frac{\sum A_{i}}{A}=\frac{\sum l_{i}\bar{d_{i}}}{A}$$ (3)

Where *A* is the total area of the image pixels, *i* is the number of fissures, *l_i_* is the length of the *i*-th fissure, and $\;\bar{d_{i}}$ is the average pixel width of the *i*-th fissure.

Relevant study indicate that the rock mass fissure ratio *K*_s_ is a valuable parameter for assessing the strength of surrounding rock integrity (Jia et al., 2001). Consequently, this paper employs the fissure ratio *K*_s_ as an evaluative criterion to classify the integrity of the rock mass at the palm surface. However, Equation 3 is not directly applicable in the image processing operation; therefore, it is reformulated based on the image characteristics, as illustrated in Equation 4.


$$K_{s}=\frac{P_{c}+P_{e}}{N}=\frac{P_{c}+P_{e}}{w\times h}$$ (4)

Where *P*_c_ and *P_e_* are the number of fissure points and edge points, respectively, *N* is the total number of pixels, and *w* and *h* are the image width and height in terms of pixel values, respectively.

The fissure number ratio depicted in the palm surface image can be derived using Equation 4. However, this equation solely accounts for the ductility of the joint fissure, neglecting the tensioning conditions affecting it. To simultaneously consider both ductility and tension conditions of joint fissures in the image, the fragmentation coefficient *K_b_* is employed to assess the integrity of the rock mass. The value of *K_b_* is defined as the sum of the fissure ratio *K*_s_ and a modified value of *K* that incorporates the tension conditions of the fissures, as calculated in Equations 5,6.


$$K_{b}=\frac{P_{c}+P_{e}}{w\times h}+K$$ (5)

$$K=\frac{2P_{c}}{P_{e}\times \sqrt{w^{2}+h^{2}}}$$ (6)

The fragmentation coefficient *K_b_* takes into account the ductility and tension of the fissures in the image, and has a good effect on the evaluation of the integrity of the surrounding rock. The integrity evaluation scheme using *K_b_* is shown in Table 8.

**Table 8:** The evaluation criteria for the fragmentation coefficient.

Fragmentation coefficient	Integrity of the tunnel palm surface	The description of tunnel palm surface
>0.15	Extremely broken	The joint fissures are dense, the main structural planes are highly open, and the rock mass is in a granular structure
0.07 ~ 0.15	Broken	There are many joint fissures, the opening degree of structural plane is high, and the rock mass is fragmented structure
0.03 ~ 0.07	Slightly broken	The number of joint fissures is general, there is a certain distance between the structural planes and the opening degree is high or general, and the rock mass fracture block structure or inlaid fragmented structure
0.01 ~ 0.03	Relatively intact	The number of joint fissures is small, there is a certain distance between the structural planes and the degree of opening is low or good, and the rock mass is medium thick layered structure or thick layered structure
<0.01	Intact	The number of joint fissures is very small, the spacing of structural planes is wide and the degree of combination is very good, and the rock mass is integral or thick layer structure

This study employs the BQ method for assessing rock quality, wherein rock body integrity serves as one of the two fundamental indicators that directly influence the classification of enclosing rock grade. The integrity of the rock mass can be evaluated using the *K_v_* value, which is derived from the number of joints per unit volume. However, obtaining the number of joints per unit volume from images poses significant challenges. Consequently, this study correlates the rock mass integrity grading, determined by the aforementioned crushing coefficient, to the *K_v_* value. The *K_v_* value is then calculated through the linear internal deviation of the crushing coefficient *K_b_* obtained from image processing, thereby establishing the essential index parameters for enclosing rock grading. The classification criteria are presented in Table 9.

**Table 9:** Correspondence between rock mass fragmentation coefficient and *K_v_* value.

Fragmentation coefficient	Rock mass integrity coefficient *K_v_*	Tunnel palm surface integrity degree
>0.15	<0.15	Extremely broken
0.07 ~ 0.15	0.35 ~ 0.15	Broken
0.03 ~ 0.07	0.55 ~ 0.35	Slightly broken
0.01 ~ 0.03	0.75 ~ 0.55	Relatively intact
<0.01	>0.75	Intact

This study also examines the influence of groundwater, which can be assessed qualitatively or quantitatively, as a correction index for perimeter rock grading. To facilitate rapid perimeter rock grading at the tunnel construction site, a qualitative approach is employed to determine the groundwater status. Consequently, this method yields the correction coefficient for groundwater, which is derived according to the qualitative evaluation method outlined in Table 10.

**Table 10:** Qualitative determination and value of groundwater.

Groundwater outflow status	BQ				
	>550	550 ~ 451	450 ~ 351	350 ~ 251	≤250
Damp or dripping water	0	0	0 ~ 0.1	0.2 ~ 0.3	0.4 ~ 0.6
Water discharge in the form of rain or linear stream	0 ~ 0.1	0.1 ~ 0.2	0.2 ~ 0.3	0.4 ~ 0.6	0.7 ~ 0.9
gushing water	0.1 ~ 0.2	0.2 ~ 0.3	0.4 ~ 0.6	0.7 ~ 0.9	1.0

Utilizing the rock hardness RC, rock integrity *K_v_*, and groundwater coefficient *K*_1_ established previously, and based on the BQ grading method, we enhance the formula for calculating the perimeter rock correction evaluation index [BQ]. This leads to the derivation of a formula suitable for the rapid real-time grading of perimeter rock quality, as calculated in Equation 7.


$$\mathrm{BW}=3R_{c}+250K_{v}+100\times (1-K_{1})$$ (7)

In the formula, *BW* is the surrounding rock classification evaluation index used in this system. Based on the calculated value, the surrounding rock grade of the working face at the current mileage is determined according to Table 11.

**Table 11:** Improved surrounding rock evaluation indices and classification.

Surrounding rock grade	*BW*
I	>550
II	550 ~ 451
III	450 ~ 351
IV	350 ~ 251
V	≤250

To validate the model’s efficacy, it was run on a laptop equipped with an Intel Core i7-12700H processor, 16 GB of RAM, and an NVIDIA GeForce RTX 3060 graphics card. The computer performed well across all performance metrics. The indicators excelled in precision evaluation. Functionally, the model achieved an accuracy rate of 95.0% and a recall rate of 96.0%. Performance testing revealed a single inference time of 0.6 milliseconds, a memory occupancy of merely 0.12 MB, and a throughput of 128,385 samples/s. In robustness testing, the accuracy rate decreased by only 3.8% when noise was added. Moreover, the model effectively handled null values, NaN, and over-range values, passing all corresponding tests. Compared with similar studies (Huang and Chen, 2023), the method proposed in this paper is faster, more practical, and more in line with engineering practice.

## 4 Engineering application of real-time classification method of tunnel surrounding rock

**Figure 16:**
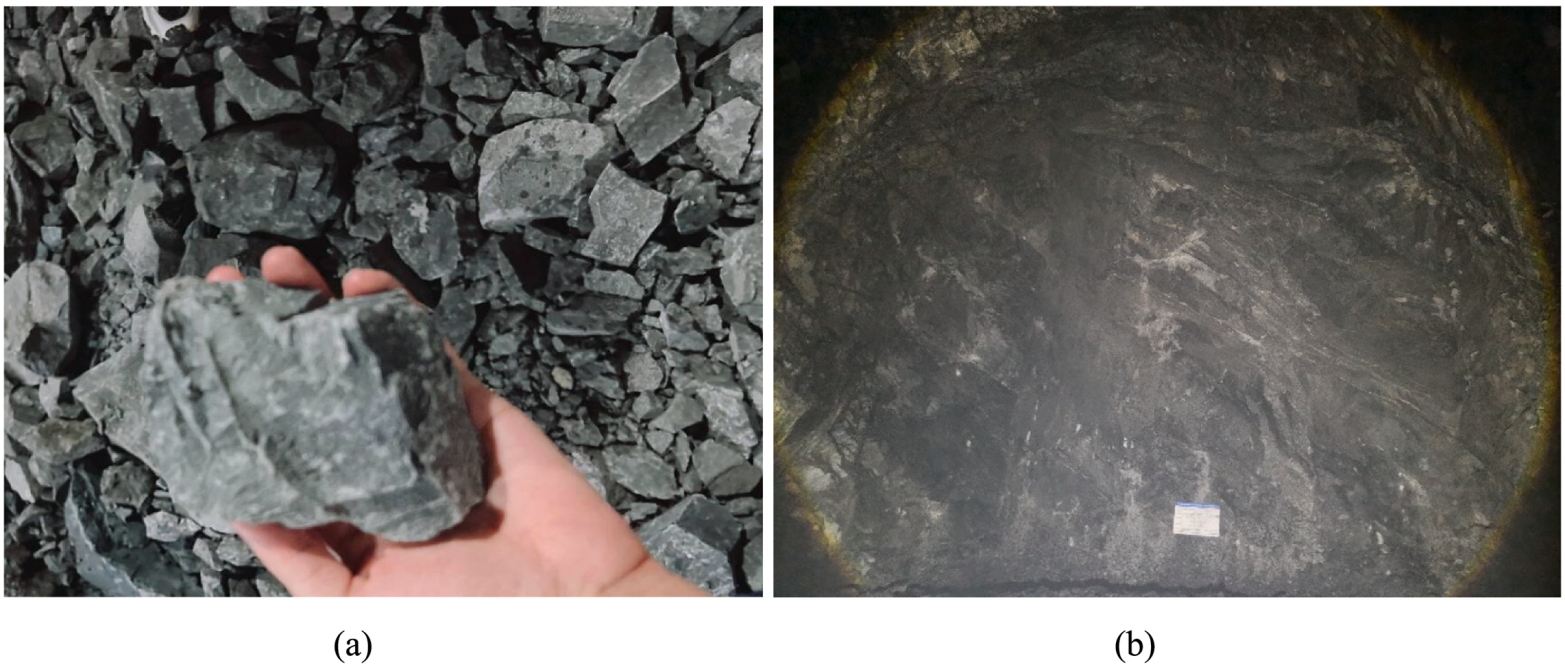
Tunnel site picture collection: **(a)** image of the rock face and **(b)** image of the tunnel palm surface.

To assess the feasibility and accuracy of this method, we utilized the key parameters of perimeter rock classification identified in the preceding section. This was complemented by the real-time perimeter rock classification model developed using the BQ method. Testing was conducted at the Xuefeng Mountain Tunnel and Jinyun Mountain Tunnel, specifically at the segments K2 + K762 to K2 + K805 in the Xuefeng Mountain Tunnel and K2 + 258 to K2 + 290 in the Jinyun Mountain Tunnel. In each tunnel, ten palm surfaces were selected for perimeter rock classification verification. Rock and palm surface images are captured using a cell phone at the tunnel site (refer to Figure 16). Uploading the images to the system for analysis and identification enables the intelligent grading of tunnel surrounding rock, a process that takes approximately 20 s. Implementing lighting equipment at the tunnel site addresses issues related to high dust levels and low light, thereby streamlining the image acquisition process and facilitating real-time rock grading. Statistical analysis is conducted on the results generated by the real-time perimeter rock grading model in comparison with both the designated perimeter rock grade and the manually determined grade, as illustrated in Table 12.

**Table 12:** Summary of surrounding rock classification results.

Tunnel name	Mileage	Assessment method	Lithology	*R*_c_	Rock hardness	Rock mass integrity	Groundwater	Surrounding rock grade
Xuefeng Mountain Tunnel	K12+625	Automated assessment	Mudstone	10.77	Slightly soft rock	Broken	Damp or dripping	V
		Manual assessment	Mudstone	—	Slightly soft rock	Broken	Damp or dripping	V
Xuefeng Mountain Tunnel	K12+627	Automated assessment	Shale	13.16	Slightly soft rock	Broken	Damp or dripping	V
		Manual assessment	Mudstone	—	Slightly soft rock	Broken	Damp or dripping	V
Xuefeng Mountain Tunnel	K12+633	Automated assessment	Mudstone	10.77	Slightly soft rock	Broken	Damp or dripping	V
		Manual assessment	Mudstone	—	Slightly soft rock	Broken	Damp or dripping	V
Xuefeng Mountain Tunnel	K12+635	Automated assessment	Mudstone	10.77	Slightly soft rock	Slightly broken	Damp or dripping	V
		Manual assessment	Mudstone	—	Slightly soft rock	Slightly broken	Damp or dripping	V
Xuefeng Mountain Tunnel	K12+638	Automated assessment	Sandstone	35.37	Slightly hard rock	Relatively intact	Damp or dripping	IV
		Manual assessment	Sandstone	—	Slightly hard rock	Slightly broken	Damp or dripping	V
Xuefeng Mountain Tunnel	K12+644	Automated assessment	Mudstone	10.77	Slightly soft rock	Slightly broken	Damp or dripping	V
		Manual assessment	Mudstone	—	Slightly soft rock	Slightly broken	Damp or dripping	V
Xuefeng Mountain Tunnel	K12+650	Automated assessment	Mudstone	10.77	Slightly soft rock	Broken	Damp or dripping	V
		Manual assessment	Mudstone	—	Slightly soft rock	Broken	Damp or dripping	V
Xuefeng Mountain Tunnel	K12+653	Automated assessment	Shale	13.16	Slightly soft rock	Broken	Damp or drippin	V
		Manual assessment	Shale	—	Slightly soft rock	Broken	Damp or drippin	V
Xuefeng Mountain Tunnel	K12+658	Automated assessment	Sandstone	35.37	Slightly hard rock	Relatively intact	Damp or drippin	IV
		Manual assessment	Sandstone	—	Slightly hard rock	Relatively intact	Damp or drippin	V
Xuefeng Mountain Tunnel	K12+670	Automated assessment	Mudstone	10.77	Slightly soft rock	Relatively intact	Damp or dripping	V
		Manual assessment	Mudstone	—	Slightly soft rock	Slightly broken	Damp or dripping	V
Jinyun Mountain Tunnel	DK25+745	Automated assessment	Mudstone	5.42	Slightly soft rock	Broken	Damp or dripping	V
		Manual assessment	Mudstone	—	Slightly soft rock	Broken	Damp or dripping	V
Jinyun Mountain Tunnel	DK25+748	Automated assessment	Mudstone	5.42	Slightly soft rock	Broken	Damp or dripping	V
		Manual assessment	Mudstone	—	Slightly soft rock	Broken	Damp or dripping	V
Jinyun Mountain Tunnel	DK25+752	Automated assessment	Shale	14.52	Slightly soft rock	Broken	Damp or dripping	V
		Manual assessment	Mudstone	—	Slightly soft rock	Broken	Damp or dripping	V
Jinyun Mountain Tunnel	DK25+758	Automated assessment	Mudstone	5.42	Slightly soft rock	Slightly broken	Damp or dripping	V
		Manual assessment	Mudstone	—	Slightly soft rock	Broken	Damp or dripping	V
Jinyun Mountain Tunnel	DK25+762	Automated assessment	Quartzite	31.12	Slightly hard rock	Slightly broken	Damp or dripping	V
		Manual assessment	Quartzite	—	Slightly hard rock	Relatively intact	Damp or dripping	V
Jinyun Mountain Tunnel	DK25+776	Automated assessment	Quartzite	31.12	Slightly hard rock	Relatively intact	Damp or dripping	IV
		Manual assessment	Quartzite	—	Slightly hard rock	Relatively intact	Damp or dripping	IV
Jinyun Mountain Tunnel	DK25+779	Automated assessment	Limestone	29.83	Soft rock	Relatively intact	Damp or dripping	V
		Manual assessment	Limestone	—	Soft rock	Relatively intact	Damp or dripping	IV
Jinyun Mountain Tunnel	DK25+785	Automated assessment	Limestone	29.83	Soft rock	Relatively intact	Damp or dripping	IV
		Manual assessment	Limestone	—	Soft rock	Relatively intact	Damp or dripping	IV
Jinyun Mountain Tunnel	DK25+792	Automated assessment	Quartzite	31.12	Slightly hard rock	Relatively intact	Damp or dripping	IV
		Manual assessment	Quartzite	—	Slightly hard rock	Relatively intact	Damp or dripping	IV
Jinyun Mountain Tunnel	DK25+795	Automated assessment	Sandstone	20.5	Soft rock	Slightly broken	Damp or dripping	V
		Manual assessment	Limestone	—	Slightly hard rock	Relatively intact	Damp or dripping	IV

The comparison of the system’s judgment results for images of 20 tunnel palm surfaces with the actual excavation geological conditions reveals an 85% accuracy rate in lithology judgment, a 75% correctness rate in rock integrity assessment, and an 80% accuracy rate in the final perimeter rock level judgment. These results satisfy the requirements for practical tunnel engineering applications, thereby validating the robustness, accuracy, and applicability of this method. Furthermore, they provide a theoretical foundation for the on-site grading and application of perimeter rock in palm surface assessments.

To investigate the causes of misjudgment in enclosure grading, field observations reveal that such misjudgments are primarily influenced by low light and high dust conditions. Consequently, it is essential to regulate the data acquisition conditions to mitigate environmental interference during the collection process.

## 5 Discussion

1. The method presented in this paper effectively addresses the challenge of real-time classification of surrounding rock in complex construction environments by integrating the lightweight neural network ShuffleNetV2 with dynamic image preprocessing techniques. In contrast to traditional deep learning models, such as ResNet and VGG, ShuffleNetV2 markedly decreases the number of model parameters through channel segmentation and depth-separable convolution, aligning with the lightweight approach of MobileNetV2 proposed by Sandler et al. (2018). Regarding image preprocessing, although the integrity assessment based on 2D images in this study has limitations in spatial connectivity analysis compared to the 3D fissure network modeling method introduced by Li et al. (2024). This constraint hinders the ability to accurately represent the 3D spatial connectivity, strike inclination, and deep extension of the fissure network. However, the method demonstrates significant engineering applicability at tunnel sites, characterized by easy data acquisition, low computational overhead, and rapid processing speed. Furthermore, it holds potential for enhancement through future integration with LiDAR or 3D reconstruction technologies.
2. In contrast to the traditional method of classifying enclosing rocks, the approach presented in this paper efficiently extracts features from images, classifies them, and significantly reduces classification time. When compared to the model developed by Liu et al. (2018), this method demonstrates greater efficiency and aligns with the robustness of the adaptive image segmentation algorithm proposed by Jiang et al. (2022). Nevertheless, the theoretical algorithm requires further refinement. Additionally, as this method does not account for the influence of groundwater, it presents certain errors and limitations when classifying surrounding rocks in water-rich tunnels. Therefore, further research is necessary to establish a classification method that incorporates multiple factors, including groundwater.
3. The comparative analysis of engineering applications demonstrates that the identification results presented in this paper align closely with the engineering site data. The accuracy of lithology, rock integrity, and enclosing rock grade exceeds 75%, thereby validating the effectiveness of this method. Although the ShuffleNet V2 neural network model utilized in this study exhibits significant efficiency and accuracy, it remains constrained by the quality of data sets, the number of training samples, and the complexity of environmental factors. Future research may establish a multifactor collaborative grading model by integrating the hydrogeological parameter fusion method proposed by Marinos and Hoek (2001), and Yuan et al. (2017), along with a migration learning strategy.
4. The field dataset for this study was primarily collected from tunnel projects in Southwest China, encompassing a range of common rock types and typical palm face morphologies. This diversity enhances the real-time grading validation for engineering applications presented in this paper. However, the limited geographic scope and specific projects from which the data were obtained result in deficiencies regarding the geological domain coverage. Rock formations in different regions may exhibit variations in lithological assemblages, joint and fissure development, weathering processes, and water-bearing conditions, all of which can influence the model’s accuracy. Consequently, future research must focus on establishing a comprehensive dataset that includes multiple regions, various types of surrounding rocks, and complex hydrogeological conditions.

## 6 Conclusion

This paper presents a real-time grading method for tunnel surrounding rock that relies on the automatic perception of parameters. An automated model for the extraction and grading of surrounding rock feature parameters is developed by integrating machine learning with image processing technologies. The primary conclusions are as follows:

1. A standard and method for collecting surrounding rock feature parameters are established. The image processing techniques employed include graying out, noise reduction, enhancement, and normalization, which collectively facilitate the efficient and accurate extraction of structural feature information from the palm surface. This approach significantly enhances the accuracy of the grading feature parameters for the surrounding rock.
2. This study employs the ShuffleNetV2 convolutional neural network to develop a model for the identification and classification of lithology, enabling rapid and precise recognition of rock types. Additionally, it proposes a method for assessing rock hardness and the integrity of surrounding rock, facilitating real-time classification.
3. Through comparative analysis at the engineering site, the feasibility and practicality of this approach are validated. The accuracy of rock property assessment is determined to be 85%, the correctness of integrity evaluation is 75%, and the accuracy of final enclosing rock classification is 80%. These results significantly enhance the efficiency and precision of enclosing rock grading.

## Data Availability

The raw data supporting the conclusions of this article will be made available by the authors, without undue reservation.
